# Trigeminal Trophic Syndrome of the Forehead: The Art of Observation in Clinical Diagnosis

**DOI:** 10.1007/s11606-025-09753-7

**Published:** 2025-07-25

**Authors:** Thomas Le, James Vu, Adrienne Atencio

**Affiliations:** Department of Internal Medicine, University of California Davis Medical Center, Sacramento, CA USA

**Keywords:** trigeminal trophic syndrome, unilateral ulceration, forehead ulcer

## Abstract

A female patient with a history of herpes zoster ophthalmicus presented with a chronic, right-sided facial ulcer consistent with trigeminal trophic syndrome (TTS), triggered by wound-picking due to concurrent anxiety and depression. TTS is a rare disorder diagnosed clinically and managed with behavioral modification alongside medical treatment.

**Supplementary Information:**

The online version contains supplementary material available at 10.1007/s11606-025-09753-7.

A 49-year-old woman with generalized anxiety disorder and depression presented with a 1.5-year history of a unilateral, non-healing, painful ulceration of the right forehead and scalp following herpes zoster opthalmicus (Fig. 1). Punch biopsy of the ulcer was negative for fungi, bacteria, active VZV, pyoderma gangrenosum or malignancy. The patient was observed picking at the wound, which played a key role in the diagnosis of trigeminal trophic syndrome (TTS). She was treated with wound care and advised against wound manipulation, which led to improved healing (Fig. 2).

**Figure 1:**
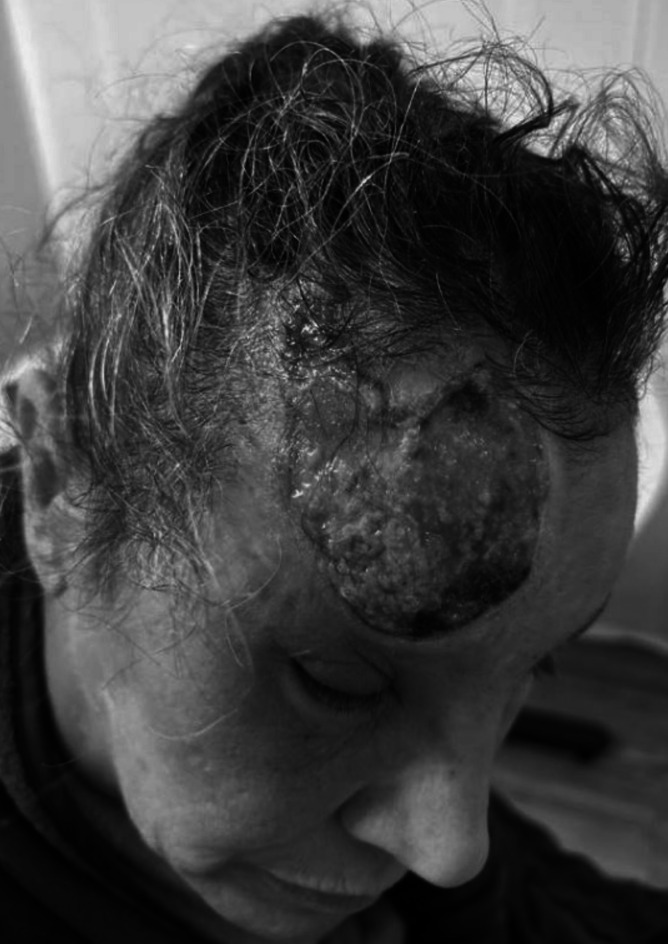
Unilateral forehead ulcer, pre-treatment

**Figure 2:**
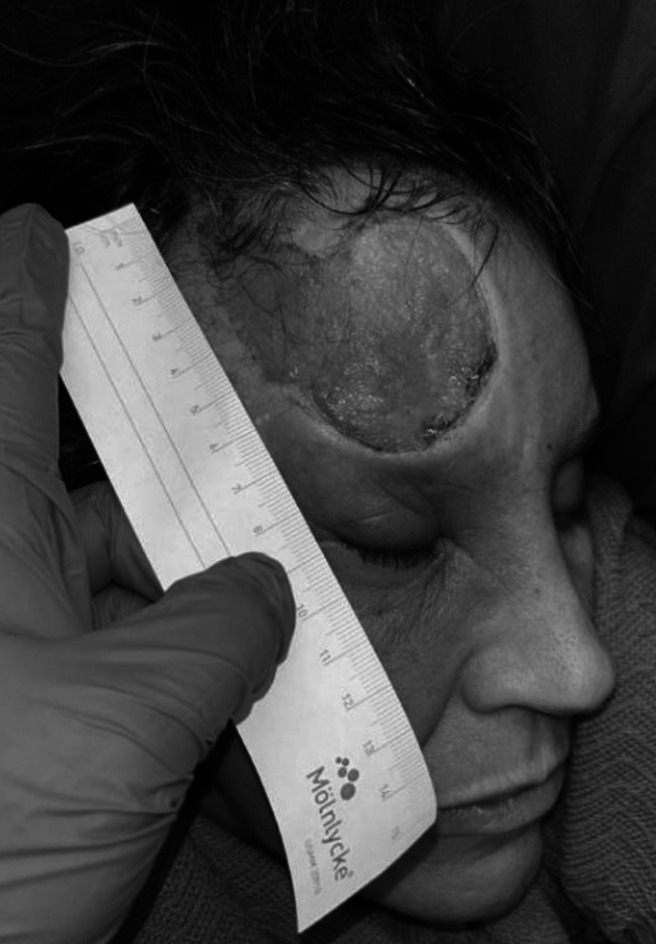
Unilateral forehead ulcer, post-treatment

TTS is a rare, clinical diagnosis of chronic ulcers resulting from self-mutilation due to dysesthesias in the trigeminal nerve distribution.^1^ The most common causes of TTS are trigeminal nerve ablation, stroke, posterior fossa tumors, trauma and herpes zoster.^2^ This case illustrates the diagnostic utility of behavioral observation. The mainstay of treatment is focused on patient education and behavior modification to prevent further self-injury. Additionally, medical management to treat dysesthesias, such as carbamazepine, pregabalin, and amitriptyline has been trialed with mixed results.^1,3,4^

## Supplementary Information

Below is the link to the electronic supplementary material.

Supplementary file1 (DOCX 72 KB)

## Data Availability

No new data were generated or analyzed in support of this research.
