# Supplementary material for: Trigeminal Trophic Syndrome of the Forehead: The Art of Observation in Clinical Diagnosis
Source: J Gen Intern Med. 2025 Jul 25;40(13):3244–7. doi: 10.1007/s11606-025-09753-7 (PMC12508317; doi:10.1007/s11606-025-09753-7)

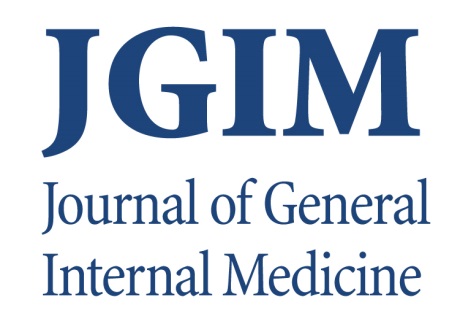


***JGIM* Statement Regarding Patient Consent**

The Corresponding Author must complete this form on behalf of all authors listed on the Title Page.

As Corresponding Author, I certify that patient permission was obtained for the use of potentially identifiable photograph(s) or other health information contained in my submission to JGIM and that the patient is aware of the context of such use.  I understand that it is my responsibility to have secured this permission and maintain the security of such personal health information.

MS#: ___________________ (if completing at first submission, leave this line blank)

Submission Title: Trigeminal trophic syndrome of the forehead: the art of observation in clinical diagnosis

Corresponding author name: Adrienne Atencio, MD

Corresponding author email address: 2315 Stockton Blvd, Suite 2P101 Sacramento CA 95817

Corresponding Author Signature:


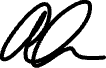


Date of Signature: 3/6/25

Patient Consent:


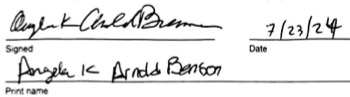

Supplement: Supplementary file 1 — Supplementary file1 (DOCX 72 KB) [file 11606_2025_9753_MOESM1_ESM.docx]
